# Carcinoma of the cervix

**DOI:** 10.1054/bjoc.2001.1759

**Published:** 2001-05

**Authors:** M Resbeut, E Fondrinier, B Fervers, C Haie-Meder, A Bataillard, C Lhommé, B Asselain, J P Basuyau, A Brémond, D Castaigne, J B Dubois, G Houvenaeghel, E Lartigau, E Leblanc, X Sastre-Garaud, F Ternier, J P Guastalla, J Chauvergne

**Affiliations:** Institut Paoli-Calmettes, Marseille; Centre Paul Papin, Angers; Centre Léon Bérard, Lyon; Cancer Research Campaign, Paris; Institut Gustave Roussy, Villejuif; Institut Curie, Paris; Centre Henri Becquerel, Rouen; Centre Val d'Aurelle Paul-Lamarque, Montpellier; Centre Oscar Lambret, Lille; Institut Bergonié, Bordeaux, France


British Journal of Cancer (2001) **84** (Supplement 2), 24–30
© 2001 FNCLCC
doi: 10.1054/ bjoc.2001.1759, available online at http://www.idealibrary.com on IDEAL®

### Carcinoma of the cervix

**M Resbeut[1], E Fondrinier[2], B Fervers[3,4], C Haie-Meder[5], A Bataillard[3,4], C Lhommé[5], B Asselain[6], JP Basuyau[7], A Brémond[3], D Castaigne[5], JB Dubois[8], G Houvenaeghel[1], E Lartigau[5], E Leblanc[9], X Sastre-Garaud[6], F Ternier[1], JP Guastalla[7] and J Chauvergne[10]**

[1]Institut Paoli-Calmettes, Marseille; [2]Centre Paul Papin, Angers; [3]Centre Léon Bérard, Lyon; [4]FNCLCC, Paris; [5]Institut Gustave Roussy, Villejuif; [6]Institut Curie, Paris; [7]Centre Henri Becquerel, Rouen; [8]Centre Val d'Aurelle Paul-Lamarque, Montpellier; [9]Centre Oscar Lambret, Lille; [10]Institut Bergonié, Bordeaux, France

Cervical cancer is the second most common cancer in women. In France, the standardized incidence in 1990 was 9.4 per 100 000. Uncommon under the age of 30, the incidence increases progressively thereafter to around 50 per 100 000 between the ages of 50 and 70 years. The standardized death rate is 3.5 per 100 000 in France and 3.9 per 100 00 in Europe.

This document covers the diagnostic and therapeutic management of microinvasive and invasive carcinomas of the cervix and excludes the screening or management of cervical intraepithelial neoplasia.

These guidelines were validated in July 1997 and will be updated according to the publication of new data.

### COLPOSCOPY

The reproducibility of colposcopy is poor and the inter- and intra-operator variability is high. The overall sensitivity and specificity of the examination is limited. The diagnosis is unreliable if the superior limit of the lesion cannot be seen, in which case endocervical curettage should be added to confirm the diagnosis (option). Because of its very poor specificity, endocervical curettage is not of value when colposcopy is satisfactory (assuming the junctional zone is seen). Colposcopy following an abnormal smear should facilitate targeted biopsies. These must be interpreted with caution, especially when destructive treatment is to follow (laser or cryotherapy) and the biopsy lesion is high-grade.

### PRETHERAPEUTIC ASSESSMENT

This must be carried out by a multidisciplinary team, which includes a specialist surgeon and a radiotherapist, irrespective of the stage of disease. An examination under general anaesthetic is optional. Cystoscopy is indicated for large-volume tumours and/or those with anterior extension (option). Rectoscopy and subsequent endorectal ultrasonography is only indicated if rectal invasion is suspected (option).

Visualization of the urinary tract is standard, using intravenous urography, renal and bladder ultrasonography, CT scanning or MRI (options). Local extension can be assessed by supra-pubic and vaginal ultrasonography or MRI (options). For proximal tumours less than or equal to stage IIB, local assessment is optional. In the absence of surgical verification, nodal extension can be assessed by CT scan, MRI or lymphography (options). There is no consensus as to which investigations should be carried out to exclude the presence of metastatic disease.

Tumour markers are not of diagnostic value. The measurement of squamous cell carcinoma antigen and of CA125 in the case of adenocarcinoma is recommended at diagnosis in order to obtain a baseline reference value. They can then be used as markers when assessing efficacy of treatment and/or progression of disease. There is no indication to measure both markers routinely (standard). Currently, there is no indication for human papillomavirus typing (standard); this should only be done in the context of a trial.

### CLASSIFICATION

The following classifications may be used (options):

- classification of the International Federation of Gynaecologists and Obstetricians (FIGO)
- TNM classification
- classification of the MD Anderson Cancer Center
- classification of the Institute Gustave Roussy.

These are all based on the local extent of tumour. The classification used most often is that of FIGO. This distinguishes limited forms (IA, IIA, IIB) and advanced forms of disease (III, IV). Within stage IIB, those with invasion proximal to the parametrium are considered as limited disease and the others as advanced disesase. In order that the results of different therapies can be compared, it is recommended that the FIGO classification is always used either alone or combined with another classification system.

### DIAGNOSIS

The histopathological examination of a surgical biopsy specimen (cone biopsy or hysterectomy) that includes the entire abnormal lesion is necessary for the diagnosis of a microinvasive carcinoma.

The histological evaluation of microinvasive carcinomas must document the (standard):

- positive diagnosis of an invasive malpighian carcinoma of limited stage
- maximum depth of tumour infiltration measured in millimetres
- degree of lateral extension of any infiltrating lesions measured in millimetres
- presence of vascular involvement
- quality of the surgical margins.

The histopathological evaluation of invasive carcinomas must include the (standard):

- confirmation of invasive cancer
- histological classification
- determination of the associated morphological risk-factors.

### PROGNOSTIC FACTORS

Independent prognostic factors are the tumour volume (for limited disease only) and the FIGO stage. The latter is correlated with local control in the pelvis, survival and the development of metastases. The prognostic value of tumour volume is limited for locally advanced disease with parametrial extension (uni- or bilateral) and for distal extension (level of evidence C). Lymphatic invasion is an independent prognostic factor for disease limited to the cervix. The number of nodes, the upper level of invasion and bilateral involvement are also of prognostic value.

### THERAPEUTIC MODALITIES

### Surgery

Staging at the time of initial surgery is standard, although if radiotherapy is to be the primary or only treatment, surgical exploration is optional. Staging includes exploration of the abdomen and lymphadenectomy (standard). Peritoneal cytology is optional and is only of value if it modifies subsequent therapy. Pelvic lymphadenectomy can be by laparotomy, by a retroperitoneal approach or by laporoscopy. Laparoscopic lymphadenectomy is only recommended if the operator has specific training in this technique. Lymphadenectomy in limited disease must include the external iliac nodes (level of evidence B). A more extensive lymphadenectomy in limited-stage disease should only be done within a trial setting.

In stage IIB disease, lymphadenectomy should be extended to the level of the renal artery before the primary tumour is excised. When the uterus is not resected, lymphadenectomy should be carried out via a retroperitoneal approach or by laparoscopy. Lymphadenectomy enables precise pretherapeutic staging of nodal involvement in order to define the extent of the radiotherapy fields necessary for treatment. The retroperitoneal route results in fewer postoperative complications related to the development of adhesions.

Ovarian transposition is also possible by laparoscopy (level of evidence B). Total hysterectomy can be by laparotomy or via the vaginal route (option). A median laparotomy is recommended for hysterectomy if the operator has no specific training in other techniques. When the ovaries are to be conserved, they must be marked with radio-opaque labels at the time of laparotomy or laparoscopy (standard). Pelvic shielding is recommended when postopertive radiotherapy is to be given. The surgeon must describe the type of uterine excision undertaken (standard) and use the classification of Piver.

### Radiotherapy

There are several techniques for endocavitary brachytherapy, including the standard system and techniques using moulds (option). Parametrial interstitial brachytherapy must only be given within a trial. Standard dosimetry is that according to the recommendations of the International Commission of Radiation Units. There is no survival advantage associated with high-dose rate compared to low dose rate delivery (level of evidence C). Brachytherapy at a low dose rate remains the standard (level of evidence C).

External radiotherapy uses photon energy equal or superior to 10 MV (standard). The use of simulation is standard. Pelvic radiotherapy should include at least two fields (standard), and the use of four fields is recommended (level of evidence C). The upper limit of the pelvic area is situated at the L4–L5 junction (standard). For low-volume stage IB–IIA disease, in the absence of nodal involvement, this limit can be reset at the level of L5–SI (option). The inferior limit depends on whether or not the vagina is involved. In all cases, the minimal safe margin is 4 cm below the lowest tumour level (standard). In the case of tumour extension to the lower vagina or of infiltration distal to the parametrium, radiotherapy must include the whole vagina (standard). The lateral limit of the antero-posterior field must always cover the nodal zones (standard). If the vagina is involved, prophylactic inguinal radiotherapy can be incorporated (option). Conformational radiotherapy, hyperthermia, neutron therapy, hyperbaric oxygen and radiosensitizers remain as research tools and must be used within therapeutic trials.

The most important factors in radiotherapy of cervical cancer are the volume irradiated, the total dose of irradiation and its duration. The sequencing of external radiotherapy and brachytherapy in radiobrachytherapy combinations has not been shown to effect results (level of evidence C). There are two options: initial external radiotherapy to the total pelvis at a minimum dose of 40 Gy followed by brachytherapy; and external radiotherapy at a lower dose preceding brachytherapy followed by a latero-pelvic boost.

For limited-stage disease, the combination of external radiotherapy/brachytherapy must deliver a total dose of 60 Gy to the central pelvis (specified by the reference isodose) and 45–50 Gy to the lateral pelvic area (standard, level of evidence C). For locally advanced disease, with distal invasion of the parametrium or metastatic pelvic nodes, a minimum of 55 Gy can be given to the pelvis (standard) with the possibility of a boost up to 65–70 Gy within very limited areas (option, level of evidence C). The central pelvic dose is also 60 Gy (as specified by the reference isodose).

The benefit of prophylactic radiotherapy to the para-aortic region (option) has not been clearly established (level of evidence C) and the risk of complications, in particular to the bowel, is greatly increased (level of evidence B). Prophylactic radiotherapy is delivered at a dose of 45 Gy (standard). For proven metastatic spread (at the para-aortic level in the absence of metastases elsewhere), para-aortic radiotherapy is standard, although the optimal dose remains to be determined.

Postoperative adjuvant radiotherapy, in the case of nodal involvement or involvement of surgical excision margins, has not been shown to be of benefit in terms of survival (level of evidence C). There is a benefit in terms of a reduction in the risk of local recurrence (level of evidence C). A variety of protocols are used and these patients should be included in therapeutic trials.

There is no evidence that modification of fractionation or schedule of radiotherapy is beneficial in this disease (level of evidence C); it should not be done outside a therapeutic trial.

### Radiochemotherapy

Concomitant administration of cisplatin with radiotherapy significantly improves local control (level of evidence A) and overall survival (level of proof B) compared with radiotherapy alone or the combination of radiotherapy and hydroxyurea. This applies to patients with poor prognosis stage IB, IIA and IIB disease

© 2001 FNCLCC 

(tumours greater than 4 cm and/or invasion of pelvic nodes and/or microscopic invasion of the parametrium) and to stage III and IVA disease. None of the patients included in these trials had para-aortic nodal involvement. Radiochemotherapy should be considered as standard treatment for these stages although the benefit seems to be less clear for patients with stage III and IVA disease (level of evidence C) and must be confirmed. The chemotherapy usually involves regimens based on cisplatin either alone or combined with 5FU.

The optimum drug scheduling has not been determined. Some regimens involve weekly cisplatin at a dose of 40 mg $m^{-2}$ and others contain doses of 50–75 mg $m^{-2}$ given every 3–4 weeks. New randomized trials should clarify what is the best schedule of chemotherapy in association with external radiotherapy or brachy therapy. The toxicity of the combination of cisplatin, 5FU brachy hydroxyurea is greater than for cisplatin alone with no greater efficacy (level of evidence B).

### Chemotherapy

Comparative trials of polychemotherapy and monotherapy have shown a non-significant increase in early response rate for polychemotherapy (level of evidence C). There is no evidence to support the use of adjuvant chemotherapy (given after surgery with or without radiotherapy) in terms of overall or relapse-free survival for patients with unfavourable prognostic factors (level of evidence C). Studies of new cytotoxics given in more efficacious, less toxic combinations are required. Intra-arterial chemotherapy remains experimental. Biological response modifiers remain within the domain of clinical research.

### Treatment toxicity

Prospective and regular long-term follow-up is recommended. The evaluation of toxicity is standard. The best system for grading treatment complications in cancer of the cervix is the Franco-Italian system of Chassagne.

The extent of surgery should not be deliberately limited in order to reduce complications unless absolutely necessary. If the true extent of disease could be determined pre-or perioperatively by new imaging techniques or by laparoscopy, surgery could be individualized, thus reducing morbidity. In this way, aggressive surgery could be limited to cases of proven extensive disease. Complications following laparoscopic surgery are less frequent, but specific training is necessary for its use.

The principle complications following treatment of cervical cancer involve the bowel and urinary tract. If the factors primarily responsible for radiotherapy-induced toxicity were known (e.g. total dose, volume of irradiation, photon energy, dose to critical organs, techniques of brachytherapy) the associated complications could be limited.

External para-aortic radiotherapy post-surgery increases the risk of gut toxicity. To limit the complications of combined radiotherapy and surgery, brachytherapy must be adapted to the surgery, and vice-versa. The dose-limiting tolerance of critical organs must be known, as stated in the recommendations of the International Commission on Radiation Units.

The toxicity of radiochemotherapy is predominantly haematological and intestinal (level of evidence B) and is greater than that of radiotherapy alone (level of evidence B). The use of chemotherapy may add to the transient side-effects of locoregional treatment in operable cancers, but should not compromise the results of surgery and contributes little to postoperative complications (level of evidence C). Induction or neoadjuvant chemotherapy before radiotherapy increases radiotherapy-induced toxicity and the risk of treatment disruption/interruption, but does not usually prevent the delivery of the planned dose (level of evidence C). It is only indicated within prospective controlled trials.

### THERAPEUTIC STRATEGY

### Stage IA disease

Surgery is standard treatment (level of evidence B). Cone biopsy (conization) or excision (amputation) of the cervix by the vaginal route necessary for diagnosis (Figure 1), is sufficient for treatment of stage IA1 disease up to 1 mm in depth (Figure 2) and from 1–3 mm (Figure 3) in the absence of bad prognostic factors (level of evidence B). If conization is to be the definitive treatment, it must be complete (standard). Conization is recommended principally for those women wishing to maintain fertility and who accept the

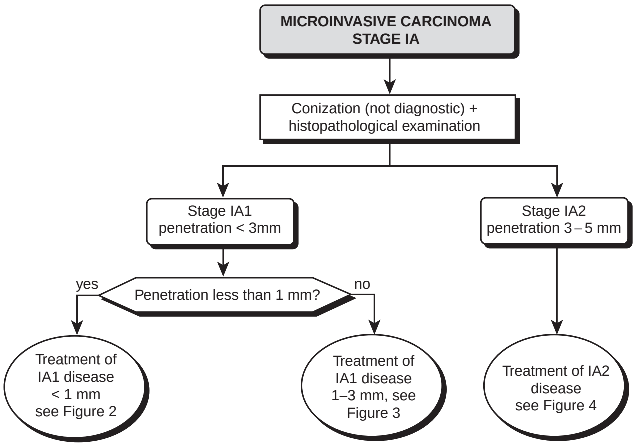

**Figure 1** Management of microinvasive carcinoma of the cervix (stage IA)

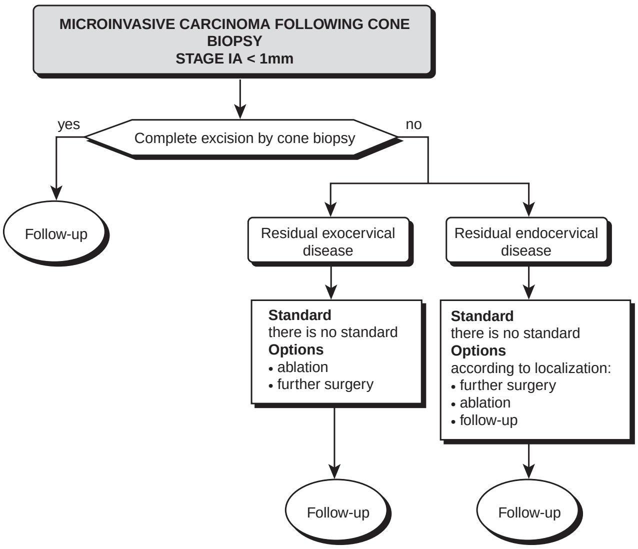

**Figure 2** Treatment of microinvasive disease (stage IA1, < 1 mm)

*© 2001 FNCLCC*

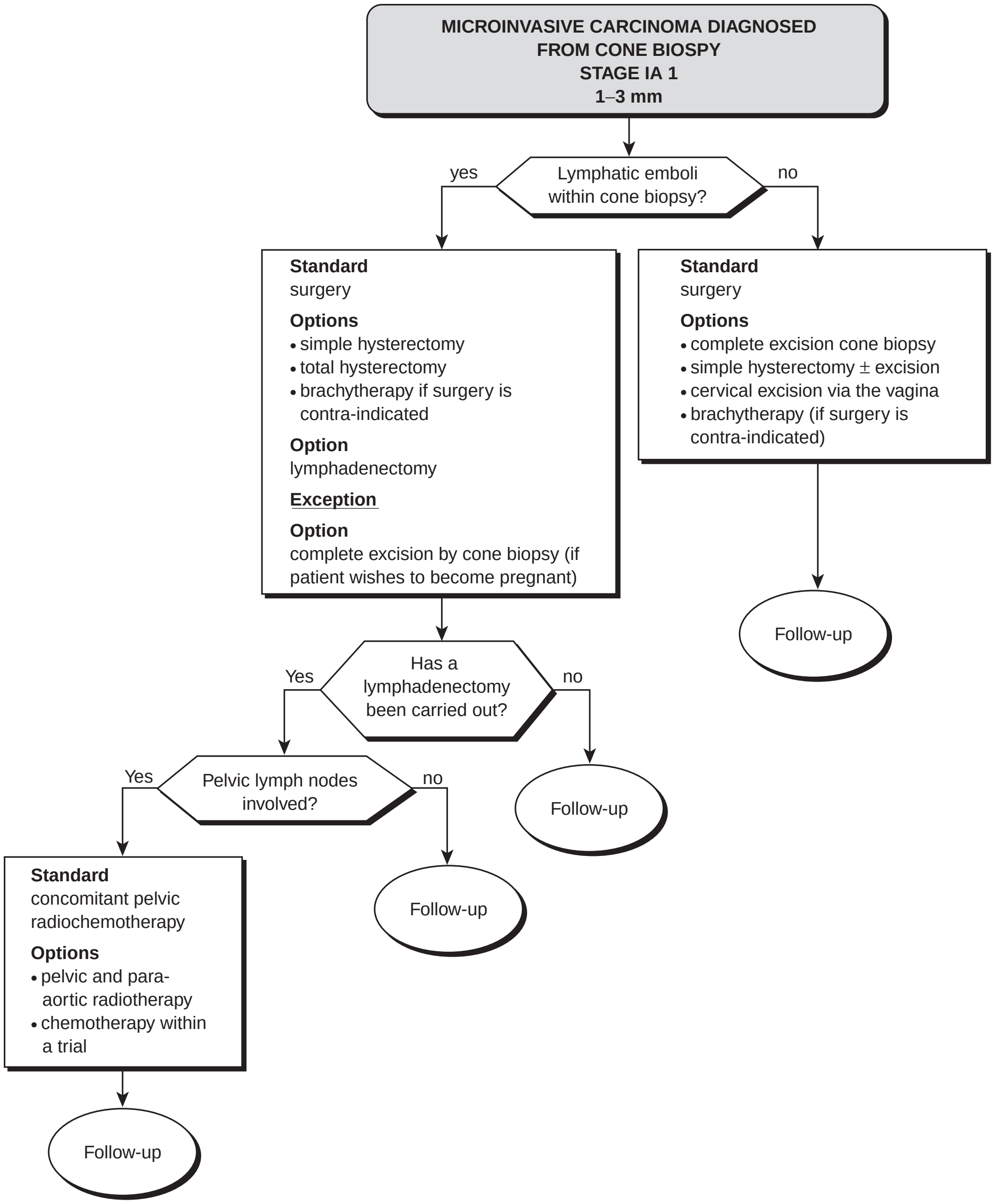

**Figure 3** Treatment of micro-invasive disease (stage IA1, 1–3 mm)

necessity for regular follow-up. A hysterectomy (Piver type I) is the other therapeutic option.

Whatever the depth of disease infiltration, the significance of lymphatic emboli as an independent prognostic factor has not been established. For disease of more than 3 mm (Figure 3), certain groups, notably the North Americans, exclude lesions with lymphatic emboli from the microinvasive category and treat them as small-stage IB1 disease. External and interiliac lymphadenectomy is recommended in the presence of lymphatic emboli. A simple or a total (Piver type II) hysterectomy are options. For disease greater than 3 mm in depth (Figure 4), (stage IA2), whether or not there are bad prognostic factors, hysterectomy is recommended. Most physicians treat these as stage IB1 disease. If there are lymphatic emboli, a total hysterectomy of Piver type II should be undertaken with external and interiliac lymphadenectomy.

### Stages IB, IIA, IIB good prognosis proximal disease

This refers to N0 tumours of less than 4 cm in size with no microscopic parametrial invasion, that have been treated surgically (Figure 5).

There is no difference in efficacy between surgery alone (Figure 6), radiotherapy alone and the combination of radiotherapy and surgery (Figure 8), irrespective of whether the tumour size is greater or less than 4 cm (level of evidence C) (Figure 5). Radiotherapy alone includes external pelvic irradiation and brachytherapy (Figure 7). Two applications of brachytherapy are given with or without a boost. Hysterectomy after a minimum delay of 8 weeks is recommended if there has been a poor response to radiotherapy, especially for tumours greater than 4 cm in size (option, level of evidence C) (Figures 7 and 9). The respective contributions of external radiotherapy (delivered to the whole

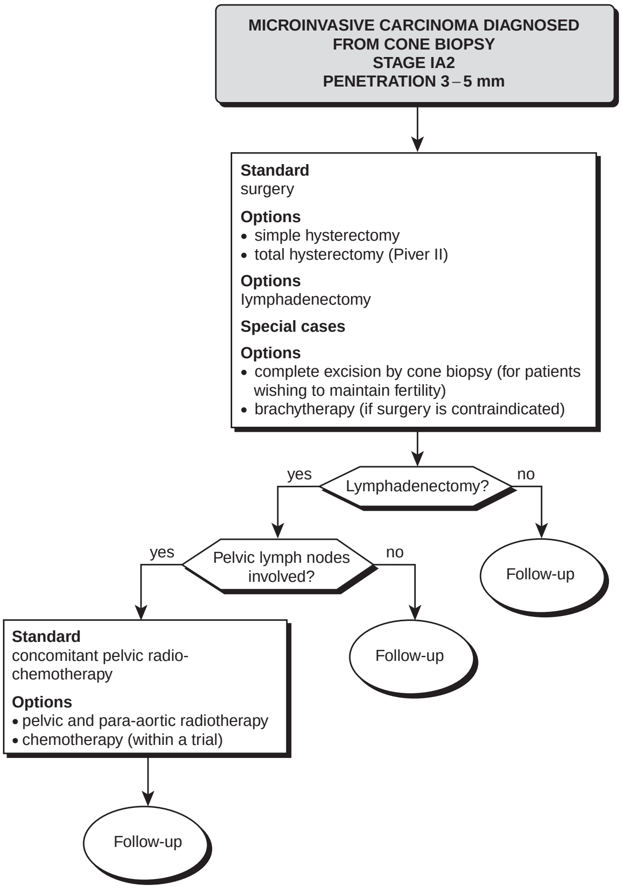

**Figure 4** Treatment of microinvasive disease (stage IA2, penetration 3–5 mm)

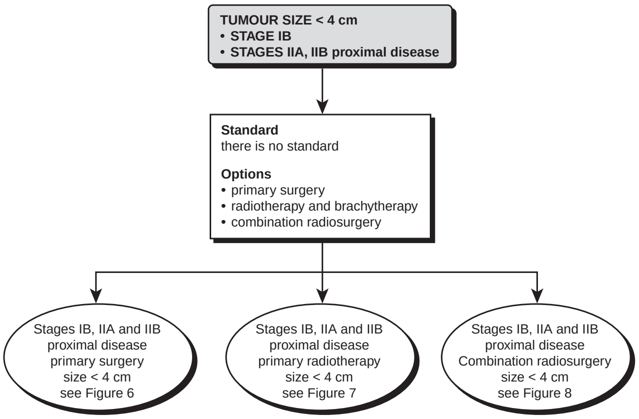

**Figure 5** Management of invasive carcinoma < 4 cm (stage IB and stages IIA, IIB proximal disease)

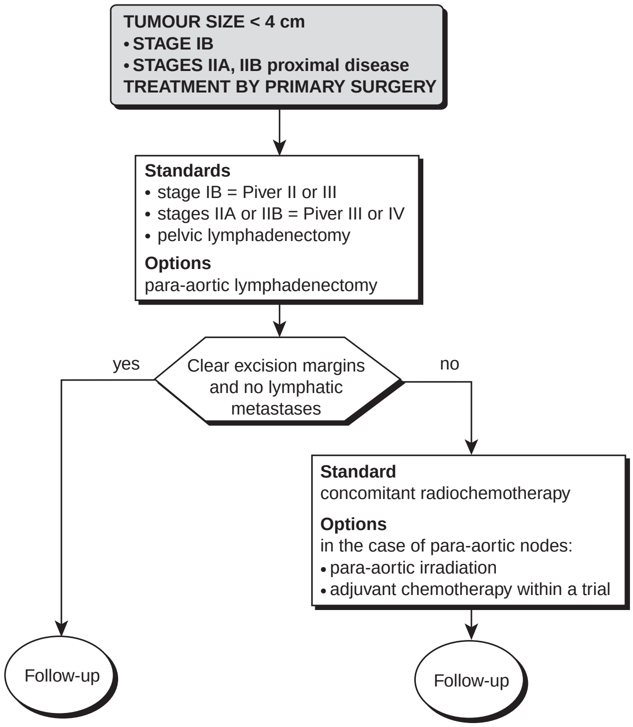

**Figure 6** Primary surgery of invasive carcinoma < 4 cm (stage IB and stages IIA, IIB proximal disease)

pelvis) and of brachytherapy (in combination with radiosurgery), depends on the size of the tumour as well as anatomic factors and the general state of the patient. The size of the dose delivered by external irradiation as compared to brachytherapy, increases with the tumour volume. When there are appropriate anatomical and clinical conditions, brachytherapy can be the sole radiotherapy treatment for tumours less than 4 cm (Figure 8). In this case, external radiotherapy is directed exclusively to the lateral pelvic regions.

### Stages IB, IIA, IIB bad prognosis proximal disease

This includes tumours greater or equal to 4 cm in size with or without invasion of pelvic nodes but without involvement of para-aortic nodes (Figure 9). Treatment can be either surgery or radiotherapy. For patients treated by primary radiotherapy, the standard is combination radiochemotherapy incorporating cisplatin. The options are: cisplatin 40 mg $m^{-2}$ per week or a cisplatin/5FU combination with 50–75 mg $m^{-2}$ cisplatin every 3–4 weeks and 4 g $m^{-2}$ FU over 4 days. There is a significant improvement in local control (level of evidence A) and overall survival (level of evidence B) following cisplatin-based combination radiochemotherapy as compared to radiotherapy alone or the association of radiotherapy and hydroxyurea.

The toxicity of radiochemotherapy is essentially that of bone marrow and gut toxicity (level of evidence B); it is greater than that associated with radiotherapy alone (level of evidence B). The best schedules of delivery of the chemotherapy have not been established. In some studies the cisplatin was given weekly at a dose of 40 mg $m^{-2}$ and in others at doses between 50 and 75 mg $m^{-2}$ every 3–4 weeks. Future randomized studies are likely to establish the optimal dose of chemotherapy to be given in combination with external radiotherapy and brachytherapy. Although of equivalent benefit, the toxicity of a cisplatin/5-FU/hydroxyurea combination is greater than that of cisplatin alone (level of evidence B).

A hysterectomy (option) after a minimal delay of 8 weeks is recommended in cases of poor response to chemotherapy, especially for tumours more than 4 cm in size (level of evidence C).

For tumours greater than 4 cm, external-beam radiotherapy is delivered first line to the whole pelvis before undertaking brachytherapy, with a boost to a total dose of 60 Gy to the lateral-pelvic

© 2001 FNCLCC

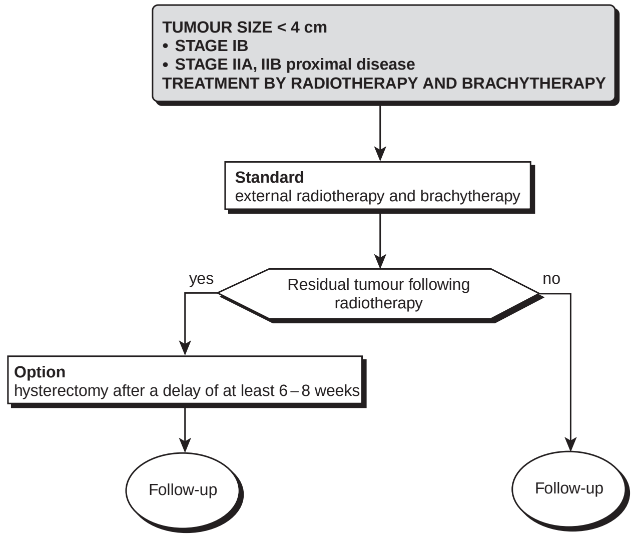

**Figure 7** Radiotherapy of invasive carcinoma < 4 cm (stage IB and stages IIA, IIB proximal disease)

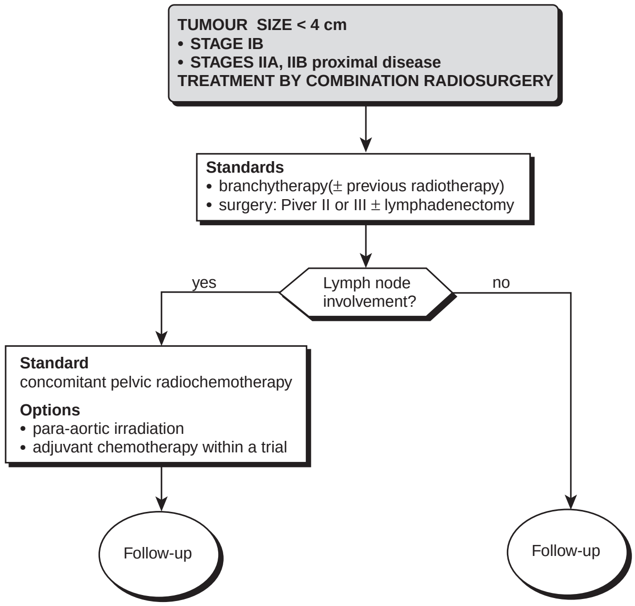

**Figure 8** Combined radiotherapy of invasive carcinoma < 4 cm (stage IB and stages IIA, IIB proximal disease)

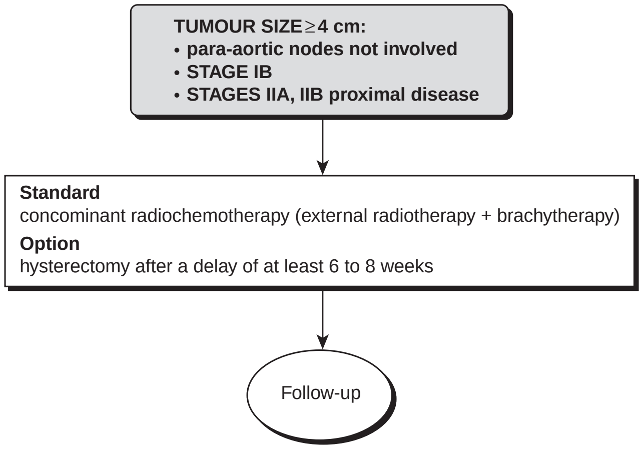

**Figure 9** Treatment of invasive carcinoma ≥ 4 cm without para-aortic node involvement (stage IB and stages IIA, IIB proximal disease)

region. Radiotherapy includes both external-beam irradiation and brachytherapy. It is recommended that the total time taken to deliver the radiotherapy (external-beam plus brachytherapy) is less than 8 weeks. The respective contribution of the external-beam pelvic radiotherapy and the brachytherapy depends on the size of the tumour and various anatomic considerations. The size of the dose delivered by external beam as compared to brachytherapy increases with tumour size. Elderly patients or those with poor performance status can be treated with radiotherapy alone.

### Stage IIB distal, stage III and IV disease

For disease beyond stage IIB with invasion distal to the parametrium, radiotherapy, either as external beam or brachytherapy, can be considered as standard treatment (Figure 10) (level of evidence C). The evidence comes from retrospective studies of patients treated with radiotherapy alone. The benefit of prophylactic para-aortic radiotherapy (option) has not been established (level of evidence C) and carries an increased risk of complications (level of evidence B). Para-aortic radiotherapy is standard treatment for proven para-aortic nodal disease in the absence of metastases elsewhere. In patients without involvement of para-aortic nodes, the combination of cisplatin-based radiochemotherapy leads to improved survival as compared to radiotherapy alone or the combination of radiotherapy and hydroxyurea (level of evidence B). This benefit is less marked for stages III and IVA disease (level of evidence C) and must be confirmed. These results have been obtained with combination chemotherapy based on cisplatin, used alone or in combination with 5-FU, but it does not appear that the combination with 5-FU gives better results than cisplatin alone (level of evidence C). The best schemes of administration of the chemotherapy remain to be established. New randomized studies are required looking as much at feasability (which is more limited in advanced forms) as toxicity of the radiochemotherapy. Pelvic exenteration is an option for stage IVA disease associated with radiotherapy and/or preoperative chemotherapy (level of evidence C). This option can be considered especially when there is no parametrial invasion, fixation to the pelvic wall, or para-aortic extension of disease. Surgery for other advanced stages (IIIB, III), with or without preoperative radiotherapy, is not recommended outside prospective therapeutic trials.

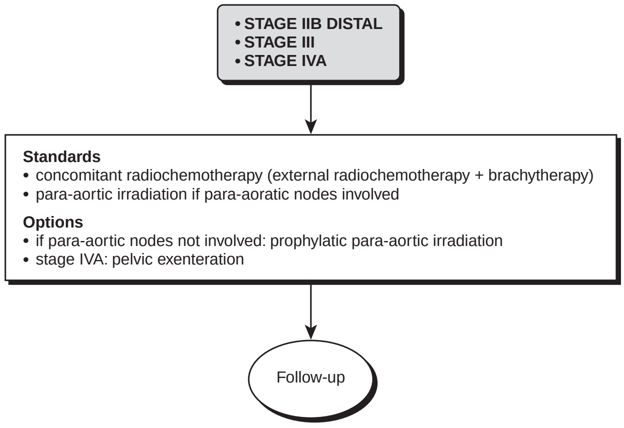

**Figure 10** Treatment of stages IIB distal, III and IV disease

*© 2001 FNCLCC*

### CANCER OF THE POST-HYSTERECTOMY CERVIX

The principle of treatment of these forms is identical to those for cancers of the cervix with an intact uterus (level of evidence C).

### CANCER OF THE CERVIX IN PREGNANCY

The recommendations for treatment are based on small series of patients. A decision to treat immediately or to defer treatment should take into consideration the stage of the tumour and the maturity of the foetus but must not compromise the chances of cure for the mother. The mother must be fully informed by a multidisciplinary team and be actively involved in the decision-making process. Stage IA disease can be followed until the end of the pregnancy. For invasive cancers, priority should be given to the treatment of the cancer during the first trimester of pregnancy. After the second trimester, the attitude towards treatment varies. Close surveillance is necessary throughout the pregnancy and treatment is reconsidered according to whether or not the tumour is progressing. The results of treatment do not appear to be affected by the state of pregnancy. As these are generally young patients, preference is often given to surgery, although there is no clear evidence to support this choice.

### FOLLOW-UP

Regular surveillance of cervical cancer after initial treatment is justified because of the possibility of treating pelvic and/or vaginal recurrence (level of evidence C). This involves history-taking and clinical examination (standard). A search for treatment-related complications should be routine (standard). The ideal frequency of follow-up has not been evaluated. The recommendations are: three to four times per year during the first 2 years, then every 6 months for the following 3 years then once a year.

The place of vaginal smears (option) in the follow-up has not been clearly established. Routine smears add nothing to clinical examination with respect to the detection of recurrence (level of evidence C) and should only be done if clinically indicated. The value of routine imaging and/or marker assays has not been demonstrated; they should only be undertaken according to clinical signs.

Hormone replacement therapy is not contra-indicated but one should be cautious with adenocarcinomas.

### PSYCHOSEXUAL ISSUES

Two thirds of patients treated for cancer of the cervix will experience psychological and sexual problems that are likely to interfere with their relationships. The prevention of sexual difficulties relies on taking precautions with respect to treatment techniques, in order to limit subsequent organic and functional problems, as well as caring for the patient’s psychological welfare. As for predictable sexual problems, the aim is to try to maintain a close and harmonious relationship between the couple. Each case must be considered individually and the exact needs of the patient considered (if possible in the presence of her partner), to select an individualized and therefore more effective management.

### INTERNAL REVIEWERS

C Cohen-Solal le Nir (Centre René Huguenin, Saint-Cloud), H Crouet (Centre François Baclesse, Caen), P Dessogne (Centre Henri Becquerel, Rouen), D Di Stefano-Louineau (Institut Paoli Calmettes, Marseille), JP Guastalla (Centre régional Léon Bérard, Lyon), A Gerbaulet (Institut Gustave Roussy, Villejuif), A Goupil (Centre René Huguenin, Saint-Cloud), S Hoffstetter (Centre Alexis Vautrin, Nancy), JC Horiot (Centre Georges-François Leclerc, Dijon), S Lasry (Centre René Huguenin, Saint-Cloud), V Le Doussal (Centre René Huguenin, Saint-Cloud), C Lhommé (Institut Gustave Roussy, Villejuif), P Martel (Centre Claudius Régaud, Toulouse), G Michel (Institut Gustave Roussy, Villejuif), J Pigneux (Institut Bergonié, Bordeaux), JF Rodier (Centre Paul Strauss, Strasbourg), L Thomas (Institut Bergonié, Bordeaux), P Troufléau (Centre Alexis Vautrin, Nancy) and B Weber (Centre Alexis Vautrin, Nancy).

### EXTERNAL REVIEWERS

C Bergeron (Institut de pathologie et de cytologie appliquées, Saint-Ouen), B Blanc (Hôpital de la Conception, Marseille), M Bolla (Faculté de Médecine de Grenoble, La Tronche), M Conte (Clinique Beauregard, Marseille), JP Dujols (Centre de Radiothérapie et d’Oncologie Médicale, Pau), L Frappart (CHU, Lyon), JP Gérard (Centre hospitalier Lyon Sud, Pierre Bénite), O Le Floch (Hôpital Bretonneau, Tours), H Lauche (Clinique Clémentville, Montpellier), JP Lefranc (Hôpital de la Pitié Salpétrière, Paris), B May (Nancy), D Querleu (CHU, Roubaix) and JM Reme (Hôpital de la Grave, Toulouse).

 © 2001 FNCLCC

